# Maternal sensitivity at the age of 8 months associates with local connectivity of the medial prefrontal cortex in children at 5 years of age

**DOI:** 10.3389/fnins.2022.920995

**Published:** 2022-09-15

**Authors:** Anni Copeland, Riikka Korja, Saara Nolvi, Olli Rajasilta, Elmo P. Pulli, Venla Kumpulainen, Eero Silver, Ekaterina Saukko, Hetti Hakanen, Eeva Holmberg, Eeva-Leena Kataja, Suvi Häkkinen, Riitta Parkkola, Tuire Lähdesmäki, Linnea Karlsson, Hasse Karlsson, Jetro J. Tuulari

**Affiliations:** ^1^FinnBrain Birth Cohort Study, Turku Brain and Mind Center, Department of Clinical Medicine, University of Turku, Turku, Finland; ^2^Department of Psychiatry, University of Turku and Turku University Hospital, Turku, Finland; ^3^Department of Psychology and Speech-Language Pathology, University of Turku, Turku, Finland; ^4^Turku Institute for Advanced Studies, University of Turku, Turku, Finland; ^5^Department of Radiology, Turku University Hospital, Turku, Finland; ^6^Helen Wills Neuroscience Institute, University of California, Berkeley, Berkeley, CA, United States; ^7^Department of Radiology, University of Turku and Turku University Hospital, Turku, Finland; ^8^Department of Pediatric Neurology, Turku University Hospital and University of Turku, Turku, Finland; ^9^Department of Pediatrics and Adolescent Medicine, Turku University Hospital and University of Turku, Turku, Finland; ^10^Center for Population Health Research, University of Turku and Turku University Hospital, Turku, Finland; ^11^Turku Collegium for Science, Medicine and Technology, University of Turku, Turku, Finland

**Keywords:** parent-child interaction, maternal caregiving behavior, maternal sensitivity, medial prefrontal cortex, functional magnetic resonance imaging, functional connectivity

## Abstract

The quality of mother–child interaction, especially maternal sensitivity in caregiving behavior, plays an important role in a child’s later socioemotional development. Numerous studies have indicated associations between poor mother–child interaction and offspring brain structure and function, but more knowledge on how variation in the characteristics of early caregiving is associated with children’s brain structure and function is needed. We investigated whether maternal sensitivity at 8 or 30 months is associated with functional connectivity in a child’s brain at 5 years of age based on the FinnBrain Birth Cohort Study (17 and 39 mother–child dyads at 8 and 30 months, respectively, with an overlap of 13 dyads). Maternal sensitivity was assessed during a free play interaction using the Emotional Availability Scales at 8 and 30 months of the children’s age. Task-free functional magnetic resonance imaging (fMRI) was acquired at the age of 5 years in 7-min scans while watching the *Inscapes* movie. Regional homogeneity (ReHo) maps were created from the fMRI data, and multiple regression analysis was performed to assess the relation between maternal sensitivity and ReHo. Maternal sensitivity at the age of 8 months was positively associated with children’s ReHo values within the medial prefrontal cortex. Distal connectivity of this region showed no significant association with maternal sensitivity in a seed-based connectivity analysis. No associations were found between maternal sensitivity during toddlerhood and brain functional connectivity. Together, these results suggest that maternal sensitivity, especially in infancy, may influence offspring brain functional connectivity. However, studies with larger sample sizes are warranted.

## Introduction

Mother–infant interaction is important for child development, especially for language, cognition, and social capabilities (Rocha et al., 2020). During infancy, while a child’s self-regulation, attachment, and cognitive skills are still developing, children are strongly dependent on caregivers, especially in their emotion regulation (Eisenberg et al., 1998). The highly sensitive period in terms of environmental impact continues to toddlerhood, which is characterized by rapid cognitive, language, and motor development (Kolb et al., 2017; Madigan et al., 2019). Positive parenting (i.e., sensitivity, support, structuring, warmth) has been shown to predict better effortful control (Neppl et al., 2020), emotion regulation (Frick et al., 2018), and executive function (Fay-Stammbach et al., 2014; Gueron-Sela et al., 2018), which are all aspects of self-regulation. On the other hand, negative parenting (i.e., intrusiveness, insensitivity) has been shown to predict depression, anxiety, and internalizing outcomes in offspring (Yap and Jorm, 2015).

Research suggests that maternal sensitivity is a key element in mother–child interaction. Maternal sensitivity refers to a mother’s ability to recognize a child’s interaction cues and emotional and physical needs and to respond to them appropriately and quickly enough from the child’s perspective. In this study, we explored maternal sensitivity using the construct of emotional availability (Biringen, 2008; Biringen et al., 2014; Saunders et al., 2015) based on attachment theory (Bowlby, 1969; Ainsworth et al., 2015) and emotional theories (Emde, 1980). Several previous studies have shown that maternal sensitivity is related to a child’s emotion regulation and reactivity in long-term development (Garvin et al., 2012; Biringen et al., 2014; Korja and McMahon, 2021). The role of maternal sensitivity changes during child development from infancy to toddlerhood, during which children acquire motor, cognitive, and language skills and start to seek autonomy. Therefore, it is crucial to study the role of maternal sensitivity during toddlerhood as well.

Current literature suggests that associations between parenting and children’s psychosocial development may be mediated by the influence of parenting on children’s brain development (Callaghan and Tottenham, 2016a). Researchers have increasingly focused on these associations between early caregiving and child brain development, yet the focus has mostly been on highly adverse experiences such as institutionalization, trauma, abuse, and neglect (Belsky and De Haan, 2011; McLaughlin et al., 2019). However, the impact of variations in typical parenting or its characteristics, such as sensitivity, on brain structure and function remains relatively poorly understood. A recent review by Farber et al. (2020) identified only 23 studies examining associations between normal variation in parenting and brain function or structure; these studies were heterogeneous in terms of research questions, methodological approaches, and findings. Another review by Ilyka et al. (2021) reviewed studies investigating the relationship between parent–infant behaviors and measures of the child’s brain structure and function; these studies showed wide variation in the neuroimaging data, while interaction data was more consistent, and maternal sensitivity was the most investigated. Previously, maternal sensitivity had been shown to be associated, for example, with hippocampal distal functional connectivity (Wang et al., 2019), hippocampal volumes bilaterally (Rifkin-Graboi et al., 2015), subcortical gray matter volume (Sethna et al., 2017), and total brain volumes (Kok et al., 2015). Finally, to the best of our knowledge, no one has examined the association between maternal sensitivity and brain local functional connectivity.

The influence of caregiving is considerable, especially in regions important to emotion regulation and cognition such as the prefrontal cortex (PFC), amygdala, and hippocampus (Callaghan and Tottenham, 2016a; Ilyka et al., 2021). During the past few decades, research has typically focused on the PFC, especially its connections with the amygdala (McLaughlin et al., 2019; Tottenham, 2020). Through bidirectional connections, the medial PFC (mPFC) is hypothesized to influence amygdala (re)activity via top-down regulation (Miller and Cohen, 2001), and there seems to be a developmental shift in these connections around the age of 10 (Gee et al., 2013; Gabard-Durnam et al., 2014). More specifically, in task-based functional magnetic resonance imaging (fMRI), amygdala–PFC connectivity switches from positive to negative around the age of 10 years (Gee et al., 2013), and in resting-state fMRI, the transition from no significant amygdala-mPFC coupling to adultlike connectivity has been shown to first emerge in those older than 10 years of age (Gabard-Durnam et al., 2014). Callaghan and Tottenham (2016b) formulated a stress acceleration hypothesis, suggesting that caregiver deprivation accelerates the maturation of the amygdala–mPFC circuit during early childhood. The strongest support for this hypothesis is from task-based fMRI studies, while most resting-state studies have suggested delayed rather than accelerated maturation in children exposed to adversity (McLaughlin et al., 2019). Yet studies linking variation in sensitivity and other aspects of parenting and amygdala–mPFC circuit connectivity are scarce and restricted to neuroimaging data acquired during middle childhood and adolescence (Thijssen et al., 2017; Kopala-Sibley et al., 2020; Jiang et al., 2021). This leaves a significant gap for studies of early childhood in which parental interactions are expected to have a particularly high impact. In addition, extant studies have focused on associations between parenting and brain distal functional connectivity, whereas local functional connectivity of the specific brain areas like PFC is poorly understood.

Our aim was to investigate associations between maternal sensitivity during infancy and toddlerhood and children’s brain functional connectivity at 5 years of age. We quantified maternal sensitivity based on a video-recorded free-play session at the ages of 8 and 30 months. At the age of 5 years, the children were scanned using task-free fMRI while they watched the *Inscapes* movie (Vanderwal et al., 2015). Effects on local brain function were explored using regional homogeneity (ReHo), followed by an analysis of longer-range connections of the affected regions using whole-brain seed-connectivity analysis (SCA). Prior studies have mostly focused on distal functional connectivity using seed-based analysis with a pre-defined seed. We decided to investigate functional connectivity with ReHo because it does not require a prior definition of the seed or region of interest (ROI) and can provide information about local connectivity throughout the whole brain. We hypothesized that maternal sensitivity is associated with ReHo in prefrontal brain areas that are known to be important to children’s emotion regulation and cognition. Additionally, we hypothesized that maternal sensitivity in infancy vs. in toddlerhood plays a more significant role for brain functional connectivity at 5 years.

## Materials and methods

This study was conducted in accordance with the Declaration of Helsinki, and it was approved by the Joint Ethics Committee of the University of Turku and the Hospital District of Southwest Finland. Written informed consent was obtained from the participants, and parents gave consent on behalf of their children.

### Participants

The participants came from the FinnBrain Birth Cohort Study, which prospectively examines the influence of genetic and environmental factors on child development and later mental and physical health outcomes (Karlsson et al., 2018). Pregnant women attending their first-trimester ultrasound were recruited by research nurses in maternal welfare clinics in the Turku region of the Southwest Finland Hospital District and the Åland Islands between December 2011 and April 2015. Ultrasound-verified pregnancy and a sufficient knowledge of Finnish or Swedish were required for participation. The cohort study includes several follow-up studies. Participants included in the present study were initially part of a follow-up of children’s neuropsychological development and parenting quality. The study design and the reasons for the choice of these measurement points and the participants of the neuropsychological measurements are covered more comprehensively elsewhere (Holmberg et al., 2022).

There were no exclusion criteria for the mother–child interaction assessments besides the original recruitment criteria for the cohort. The exclusion criteria for the neuroimaging visit were: (1) born before gestational week 35 (week 32 for those with exposure to maternal prenatal synthetic glucocorticoid treatment), (2) developmental or major organ abnormalities in senses or communication (e.g., blindness, deafness, congenital heart disease), (3) known long-term medical diagnosis (e.g. epilepsy, autism), (4) ongoing medical examinations or clinical follow-up in a hospital, (5) the child using continuous, daily medication (including oral medications, topical creams, and inhalants; desmopressin was allowed), (6) history of head trauma (defined as concussion necessitating clinical follow up in a health care setting), (7) metallic ear tubes, and (8) routine MRI contraindications. Obstetric data were retrieved from the Finnish National Birth Register (National Institute for Health and Welfare), and other background information was gathered using questionnaires.

In total, 197 families participated in the 8-month assessment and 415 in the 30-month assessment; 203 children participated in a neuroimaging visit at 5 years of age, and 77 of them had successful functional scans. The current sample included all children with fMRI data at the age of 5 years and interaction data from at least one age point, defining a final cohort of 17 mother—child dyads with mother—infant interaction data at the age of 8 months and 39 mother—child dyads at the age of 30 months. Of the included dyads, 13 took part in interaction assessments at both age points. There were no statistically significant differences between groups in the background variables. The sociodemographic characteristics of the samples are presented in Table 1.

**TABLE 1:** Demographics of the mother–child dyads included in this study.

	Mother–infant dyads (*N* = 17)	Mother–toddler dyads (*N* = 39)
**Categorical variables**	***N*** **(%)**	***N*** **(%)**
**Child sex**		
Male	6 (35.3)	13 (33.3)
Female	11 (64.7)	26 (66.7)
**Maternal background**		
Finnish	17 (100)	39 (100)
Other	0 (0)	0 (0)
**Parity**		
Primiparous	9 (52.9)	20 (51.3)
Multiparous	8 (47.1)	19 (48.7)
**Maternal smoking during pregnancy**		
No smoking	16 (94.1)	39 (100)
During first trimester	1 (5.9)	0 (0)
During third trimester	0 (0)	0 (0)
**Maternal education level**		
Upper secondary school or vocational school or lower	5 (29.4)	9 (23.1)
University of applied sciences	3 (17.6)	9 (23.1)
University	9 (52.9)	21 (53.8)
**Maternal monthly income (euros)**		
≤1,500	4 (23.5)	9 (23.1)
1,501–2,500	12 (70.6)	23 (59.0)
2,501–3,500	0 (0)	6 (15.4)
≥3,501	1 (5.9)	0 (0)
Missing	0 (0)	1 (2.6)
**Continuous variables**	**Mean (SD) [range]**	**Mean (SD) [range]**
Maternal sensitivity (EAS)	5.2 (1.4) [2–7]	5.2 (1.1) [3–7]
Duration of gestation (weeks)	39.7 (1.6) [36–42]	39.9 (1.3) [36–42.3]
Birth weight (grams)	3552.1 (526.5) [2,530–4,900]	3547.0 (488.4) [2,530–4,900]
Child age at interaction (months)	8.0 (0.4) [7.4–8.7]	30.1 (0.4) [29.5–31.4]
Child age at interaction, corrected for GA (months)	8.0 (0.2) [7.8–8.3]	30.1 (0.5) [29.1–31.1]
Child age at scan (years)	5.4 (0.1) [5.3–5.8]	5.4 (0.1) [5.3–5.8]
Child age at scan, corrected for GA (years)	5.4 (0.1) [5.3–5.7]	5.4 (0.1) [5.3–5.7]
Maternal age at childbirth (years)	31.1 (4.7) [21–39]	30.9 (4.6) [23–41]
Maternal pre-pregnancy BMI (kg/m^2^)	24.2 (4.7) [17.5–34.4]	23.6 (4.3) [17.5–34.4]
**Maternal EPDS score**		
Gestational week 34	6.6 (6.4) [0–20]	4.4 (4.5) [0–19]
3 months postpartum	4.6 (3.6) [0–12]	3.5 (3.4) [0–12]
6 months postpartum	7.1 (5.8) [0–19]	4.8 (4.4) [0–19]

There were no statistically significant differences between groups in the background variables. SD, standard deviation; EAS, Emotional Availability Scale; GA, gestational age; BMI, body mass index; EPDS, Edinburgh Postnatal Depression Scale.

### Measures

#### Maternal sensitivity

Mother–child interaction assessments were conducted as part of the Child Development and Parental Functioning Lab study visits when the children were aged 8 and 30 months. The mothers were instructed to play with their children the way they were used to, either with or without toys. The 20-min (8 months) and 15-min (30 months) free-play sessions were videotaped and later coded using the Emotional Availability Scale (EAS) 4th Edition (Biringen, 2008). The EAS is comprised of four parental (sensitivity, structuring, non-instrusiveness, and non-hostility) and two child (responsiveness and involvement) dimensions. In this study, we used the sensitivity dimension. Maternal sensitivity consists of a mother’s behaviors and emotions that create and maintain a healthy and positive connection with her child, including how appropriately and promptly mothers meet the physical and psychological needs of the child.

All dimensions consisted of seven subscales scored on a 3- or 7-point scale to yield a total score from 7 to 29. The dimensions were also scored on a Likert-type scale to yield a direct score of 1-7 reflecting the evaluator’s overall view of the emotional availability. The coding was performed by two (at 8 months) and three (at 30 months) blinded, trained, and reliable coders. Interrater realibility was assessed for 10% of the videotapes. Intraclass correlation coefficient were 0.80 for sensitivity at 8 months and ranged from 0.83 to 0.91 at 30 months. Differences were negotiated between the coders. The direct score of maternal sensitivity was used as the continuous variable in the present study.

#### Magnetic resonance imaging scanning visits

All fMRI scans were performed for research purposes, and participants were scanned awake and without sedation. The imaging was performed at the Department of Radiology, Turku University Hospital between October 2017 and March 2021.

Before scanning, a research staff member met with the families personally. Parents were advised to use familiarization methods, such as reading a story and showing a video describing the visit, playing audio of scanner sounds, encouraging the child to lie still like a statue, and practicing with a homemade mock scanner, e.g., a cardboard box.

On the scanning day, imaging was practiced with a wooden mock scanner using the child’s own toy to “scan,” and the effects of motion were illustrated by taking sharp and blurry pictures using a mobile phone camera. Participants were shown the *Inscapes* movie beforehand on a tablet screen. At the end of the preparation, a light meal was served. The preparation phase lasted 1–2 h.

To increase comfort and minimize head motion, foam paddings were positioned around the participant’s head inside the head coil. A leg cushion under the knees and customized weighted blanket were provided if desired. Earplugs (Mack’s Soft Moldable Silicone Putty Earplugs) and MRI safe headphones (Siemens Medical Solutions) were provided for noise attenuation. A parent and a member of the research staff stayed in the imaging room right next to the child throughout the whole session. Research personnel continuously monitored the scanning in the scanning room and from the control room through a window with a microphone contact.

#### Image acquisition

MRI scans were conducted on a Siemens Magneton Skyra fit 3T scanner (Siemens Medical Solutions, Erlangen, Germany). A 20-element Head/Neck Matrix coil allowed the use of the generalized autocalibrating partially parallel acquisition (GRAPPA) technique to accelerate acquisitions (parallel acquisition technique [PAT] factor of 2). The scans included a high-resolution T1 magnetization-prepared rapid gradient echo (MPRAGE), a T2 turbo-spin echo (TSE), diffusion tensor imaging (DTI), and a 7-min fMRI. The fMRI consisted of 170 volumes with voxel size 3.0 × 3.0 × 3.0 mm, TR 2,500 ms, TE 30.0 ms, flip angle of 80°, and 42 axial slices without gaps. Full cerebellar coverage was not possible in all participants. Prior to fMRI acquisition, all children had rested by watching a movie or a TV show of their choice during the 30–45 min required for structural scanning. If the child had fallen asleep, they were gently awakened. During the fMRI sequence, participants were instructed to stay as still as possible with their eyes open. To minimize motion and reduce cognitive load, the *Inscapes* movie was played during fMRI data collection (Vanderwal et al., 2015). Visual stimuli were presented on an MRI-compatible 32” LCD monitor with full HD resolution (Nordic Neuro Lab) located at the foot of the bed of the scanner, where participants could watch via mirrors mounted on the head coil. The total scanning time was limited to 1 h, and the imaging was discontinued if the child expressed unwillingness to continue at any point.

Anatomical images were screened by an experienced neuroradiologist (author RP) for incidental findings (Kumpulainen et al., 2020). None of the participants included in the present study had clinically relevant incidental findings.

### Image analysis

#### Regional homogeneity

Functional MRI data were slice-timing corrected and motion corrected in the FMRIB Software Library (FSL; Jenkinson et al., 2012) v6.00 relative to a manually chosen reference volume, free of major artifacts. Motion outliers were estimated using artifact detection tools (ART).^1^ We tagged the images as outliers if they had composite motion threshold > 2 mm or DVARS > 9, which are default parameters in the ART toolbox and worked well for our current data. All children included in the final statistical analyses had a full fMRI sequence of 170 volumes, and a maximum of 50 volumes were tagged as outliers by ART. The descriptive statistics for motion were as follows: motion outliers (mean 12, range 0–44), mean absolute displacement (mean 0.51, range 0.11–1.81, mm), and mean relative displacement (mean 0.21, range 0.03–0.83, mm). Anatomical masks for white matter and cerebrospinal fluid were defined in the Montreal Neurological Institute (MNI) standard space and registered to functional data with an affine transformation. Average signal in white matter and cerebrospinal fluid as well as 24 motion covariates (the six realignment parameters and their temporal derivatives and quadratic terms) were included as nuisance covariates. Taken together, denoising consisted of outlier rejection, nuisance regression, detrending, and high-pass filtering (0.008 Hz).

The main derived brain metric was ReHo, which was estimated in a data-driven manner and provided a voxel-wise local connectivity measure across the whole brain. ReHo is based on calculating the Kendall’s coefficient of concordance over a target voxel and neighboring voxels. ReHo was computed as implemented in DPABI (number of voxels in a cluster; *N* = 27).^2^ For group analysis, ReHo maps were normalized non-linearly using FSL FNIRT to 1.0 × 1.0 × 1.0 mm^3^ MNI space. Finally, the data were smoothed with a Gaussian filter of 6 mm full width at half maximum (FWHM).

### Seed-connectivity analysis

In line with our prior work (Rajasilta et al., 2021), we had predefined plans to conduct complementary exploratory SCAs guided by the ReHo results. The SCA analyses were performed with FSL tools using the same preprocessing and nuisance regression as for the ReHo analyses. The seed ROI was defined by a 3-mm-radius sphere generated in FSL’s FSLeyes and corresponding to the location of our ReHo result, the cluster peak MNI coordinate (-6, 44, 28). The average time series of the seed ROI was extracted from normalized data using the “fslmeants” command. Subject-level seed-based connectivity maps were generated using the FSL v6.00 fMRI expert analysis tool (FEAT; Woolrich et al., 2001). The resulting z-score maps for each participant were then normalized to MNI space, and statistical tests were conducted with FSL FEAT higher-level analysis.

### Covariates

The following covariates were included: child sex, child age at fMRI (days), child gestational age (weeks), maternal age at childbirth (years), maternal pre-pregnancy body mass index (BMI) (kg/m^2^), maternal smoking during pregnancy, maternal education level, and perinatal maternal depressive symptoms. Smoking during pregnancy was based on maternal self-report at the first and third trimesters. Education level was categorized into three classes: upper secondary school or vocational school, university of applied sciences, and university. Maternal depressive symptoms were measured at gestational week 34 and 3 and 6 months postpartum using the Edinburgh Postnatal Depression Scale (EPDS; Cox et al., 1987), and the summed scores were used as a covariate.

### Statistical analysis

Demographic data was analyzed with IBS SPSS Statistics (version 27). ReHo analyses were conducted with general linear models (GLMs) via the “Multiple Regression” option of SPM12 (running on MATLAB 2016b).^3^ Maternal sensitivity was set as the main explanatory variable, and child age and sex were set as independent variables of no interest. The *a priori* threshold for voxel-level statistical significance was set to *p* < 0.005 and a family-wise error (FWE) correction at the cluster level to *p* < 0.05. We also systematically tested whether the results survived a more stringent threshold at *p* < 0.001.

For visualization, we plotted the association between the mean ReHo values of previously identified clusters of ReHo analysis and maternal sensitivity scores in SPSS Statistics. To assess the sensitivity of the association between ReHo and maternal sensitivity to possible confounding factors, we first calculated the partial correlation in line with the SPM model, that is, we explained the ReHo values in the frontal cluster with maternal sensitivity and the main explanatory variable and child age and sex as independent variables of no interest. We then repeated the partial correlation analysis while additionally controlling for maternal age at childbirth, pre-pregnancy BMI, smoking during pregnancy, education level, perinatal depressive symptoms, and child gestational age. Of note, we regard the SPM models as our main finding and report the effect sizes of the partial correlations to demonstrate any potential effects of additional confounding factors, being careful not to perform double dipping to guide the interpretation of the results (Kriegeskorte et al., 2009). There were a few (1–2) missing EPDS scores depending on the timepoint used, and they were handled as missing data, that is, no imputation was performed.

FSL GLM models’ one-sample *t*-tests were used to determine mean seed-based connectivity from subject-level SCA maps. Additionally, an FSL GLM regression model was used to examine associations between subject-level SCA maps and maternal sensitivity, with child sex and age as covariates. We limited the analyses to gray matter by using an inclusive gray-matter mask that included both cortical and subcortical regions.

## Results

### Associations of maternal sensitivity at 8 months with regional homogeneity at 5 years of age

**FIGURE 1:**
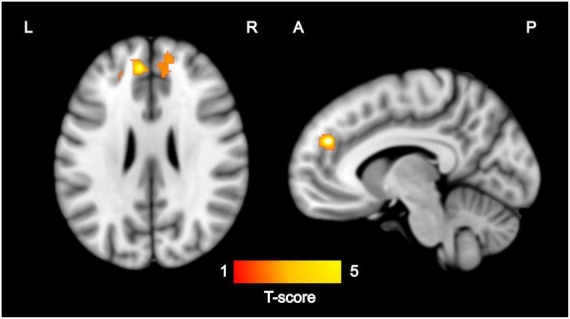
Regions where ReHo values were significantly (*p* < 0.005, *p* < 0.05 FWE-corrected) correlated with maternal sensitivity during infancy (*N* = 17). Images are displayed on the MNI template in sagittal and axial slices. Color bar represents Z-scores. L, left; R, right; A, anterior; P, posterior; ReHo, regional homogeneity; FWE, family-wise error; MNI, Montreal Neurological Institute.

Among the 17 mother–child dyads, the multiple regression analysis for ReHo and maternal sensitivity during infancy showed a positive association [*p* < 0.005, *p* = 0.027 FWE-corrected, cluster size (kE) 704] with the mPFC. Based on the automatic anatomic labeling atlas, the peak cluster (-6, 44, 28) was located in the left medial superior frontal gyrus (SFGmed. L) with extensions to the right medial superior frontal gyrus (SFGmed. R) and right anterior cingulate and paracingulate gyri (ACG. R) (Figure 1). There were no negative associations between maternal sensitivity and ReHo maps at *p* < 0.005.

**FIGURE 2:**
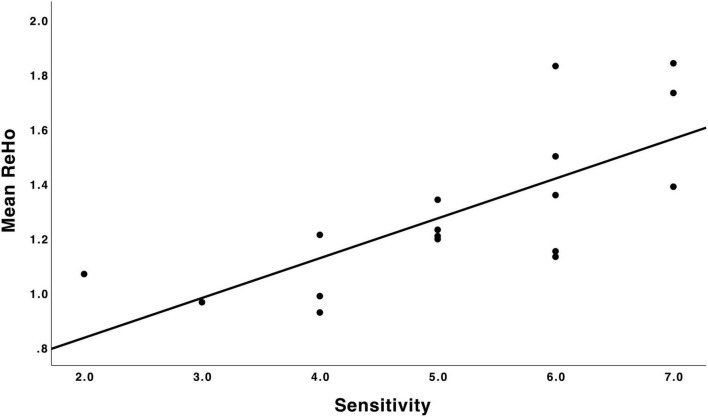
Scatter plot depicting the relation between maternal sensitivity during infancy (*N* = 17) and mean ReHo in the mPFC. A higher maternal sensitivity score was associated with higher regional homogeneity in the mPFC. ReHo, regional homogeneity; mPFC, medial prefrontal cortex.

For sensitivity analyses, we estimated a mean ReHo value from the peak cluster across 704 voxels. There was a positive partial correlation between mean ReHo values (mean 1.29, SD 0.28) and maternal sensitivity scores (mean 5.18, SD 1.42), which was statistically significant (*r* = 0.80, *p* < 0.001, corrected for child sex and age at scan). Mean ReHo values and maternal sensitivity scores are presented in a scatter plot in Figure 2. In the sensitivity analyses, the effects on the correlation map of ReHo and maternal sensitivity survived as statistically significant (*r* = 0.88 and *p* = 0.048 with all chosen covariates added into the model). We also ran sensitivity analyses in SPM by adding each of the variables used in the partial correlation models individually as an additional covariate of no interest (1 variable at a time). These analyses revealed that the frontal cluster that we report as our main finding is consistently identified, but most of the models do not survive the *a priori* threshold *p* < 0.005 at the cluster-corrected level *p* < 0.05, which is likely due to small sample size (see “Limitations” section).

**FIGURE 3:**
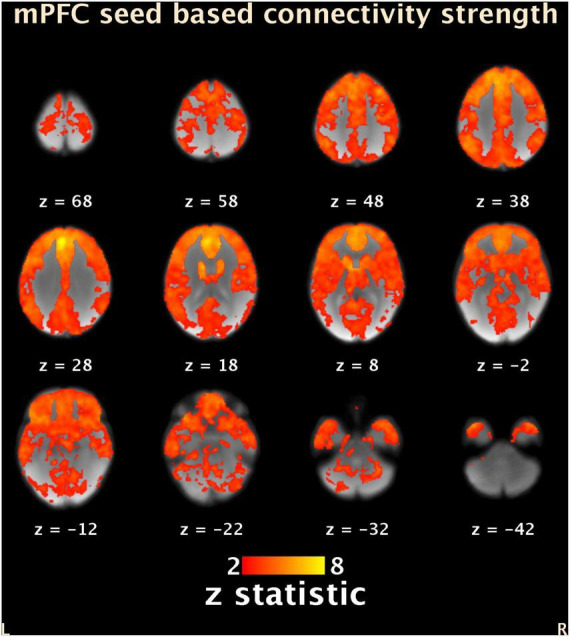
Group average (*N* = 17) seed-based connectivity map of the mPFC (-6, 44, 28). Results are masked with the same gray matter mask as for the ReHo analyses. Locations of the axial slices are given according to MNI space. Color bar represents Z-scores. L, left; R, right; mPFC, medial prefrontal cortex; ReHo, regional homogeneity. MNI, Montreal Neurological Institute.

Group mean SCA showed that time series in the mPFC seed showed positive correlations in widespread brain regions (Figure 3). However, no statistically significant effects were revealed from multiple regression analysis between functional connectivity and maternal sensitivity.

### Associations of maternal sensitivity at 30 months with regional homogeneity at 5 years of age

Analyses of the 39 mother–toddler dyads showed no statistically significant associations between maternal sensitivity during toddlerhood and ReHo maps at the age of 5 years at *p* < 0.005.

## Discussion

In the present study, we found that maternal sensitivity in infancy is associated with local functional connectivity of medial prefrontal areas of a child’s brain at the age of 5 years. These prefrontal regions showed widespread connectivity to frontal areas and across the brain, but the strength of these longer-range connections was not significantly associated with maternal sensitivity. Further, there was no association between maternal sensitivity in toddlerhood and child brain connectivity. To the best of our knowledge, this is the first study examining associations between maternal sensitivity toward children in the age range of 8–30 months and brain task-free functional connectivity at the age of 5 years. Although the study was limited by its small sample size, the results provide preliminary evidence about the importance of maternal sensitivity in infancy for later functional brain connectivity.

The PFC is one of the key brain regions related to emotional and cognitive regulation (Miller, 2000; Kolb et al., 2012; Dixon et al., 2017), and it is known to be sensitive to environmental influences. Altered functional connectivity of the PFC is commonly reported in neuropsychiatric disorders, such as attention deficit hyperactivity disorder (Zang et al., 2007) and autism spectrum disorders (Doyle-Thomas et al., 2015), which typically involve problems in self-regulation and executive function. In the present study, we found that maternal sensitivity during infancy was associated with higher ReHo in the PFC. A higher ReHo value represents higher coherence and centrality, i.e., higher local synchrony of brain activity (Zang et al., 2004). The normative developmental trajectory of local connectivity shows general reduction in ReHo with age, representing a transition from local to distributed organization (Lopez-Larson et al., 2011). In line with this idea, our finding that ReHo values in the mPFC were higher in children with higher maternal sensitivity is suggestive of amplified local and, conversely, still immature distal connectivity of the mPFC.

We found no associations between children’s mPFC distal connectivity and maternal sensitivity. When mature, the PFC shows widespread structural and functional connections across the brain (Kolb et al., 2012; Sousa et al., 2018). Generally, the adult-like whole-brain connectivity of the PFC is known to be late-emerging (Pujol et al., 2021), but some evidence suggests that the PFC undergoes rapid development during infancy (Hodel, 2018). Our results showed evidence that the PFC’s functional connectivity is already established in early childhood (Figure 3). However, we did not find associations between connectivity and maternal sensitivity. The reasons this broad connectivity was not related to maternal sensitivity may lie in the small sample size and relatively low variability in maternal sensitivity scores in the sample. It is also possible that the distal connectivity between the mPFC and other brain areas sensitive to parenting influences are still immature at the age of five, and the possible programming effects of parenting do not appear yet at this age point. Previous studies showing associations between parenting and mPFC distal connectivity have mostly focused on middle childhood and adolescence (Thijssen et al., 2017; Kopala-Sibley et al., 2020; Jiang et al., 2021), so further research covering early childhood is required.

Further, although we found an association between maternal sensitivity during infancy and local functional connectivity in the mPFC at the age of 5 years, we did not find similar associations when maternal sensitivity was investigated during toddlerhood, even though the sample size at this age was bigger. These findings add to the research on infancy representing a highly sensitive period in brain development and, more specifically, PFC development and are consistent with previous behavioral research showing that mother–infant interaction plays a key role in children’s psychosocial development (Perry et al., 2017). The effects of maternal sensitivity during toddlerhood were not significant, which might be explained by parenting having a different role in toddlerhood in comparison to infancy (i.e., the heightened need for structuring and behavioral control in addition to aspects of sensitivity).

Although the role of parenting might change when a child develops from infancy to toddlerhood, it has been shown that elements in mother–child interaction, especially maternal sensitivity, are moderately stable throughout infancy and toddlerhood (Célia et al., 2018; Holmberg et al., 2022). Because brain structure or function was not assessed at baseline concurrently with interaction assessment, the effects of parenting detected in the current study could be due to a baseline association that has not been accounted for. Consequently, longitudinal imaging data covering the earlier ages as well are needed to better understand the timing of the effects on brain development and examine change in brain structure and function over time (Bhanot et al., 2021). Finally, it would be important to link the findings regarding associations between parenting and children’s brain structure and function with children’s behavioral and cognitive outcomes to better understand the role of the identified associations in child development.

This study has several limitations. First, our sample is small, which limits the generalizability of the findings and calls for future studies guided by our preliminary observations. Regarding this limitation, the number of participants is, in general, low in cognitive neuroscience and neuroimaging studies (Szucs and Ioannidis, 2020), and this is known to possibly artificially inflate correlations (Vul et al., 2009). Recently, Marek et al. (2022) showed how brain–behavior correlations can range from −0.52 to 0.52 in samples of *N* = 25 just due to sampling variability. Additionally, Szucs and Ioannidis (2020) computed a minimum of 34 participants to surpass 80% power to detect an effect size of D = 0.5 at a threshold of 0.05. However, the dilemma of small sample sizes and low statistical power depending on sample size concerns the entire neuroimaging research field, including the most highly cited studies (Szucs and Ioannidis, 2020). Second, our sample is unbalanced between sexes. Third, although we measured maternal sensitivity at two different timepoints, at 8 and 30 months, the assessments were performed on separate samples with only partial overlap (*N* = 13). These factors may have affected generalizability. Furthermore, we did not examine different aspects of mother–child interaction, as we wanted to focus on maternal sensitivity, which is known to be a key element in mother–child interaction based on previous literature. We also used a passive viewing paradigm during fMRI acquisition to reduce head motion (Vanderwal et al., 2019; Finn and Bandettini, 2021), which is a significant challenge in pediatric fMRI research. There was still considerable motion in the data, which may have affected our results. Similarly, the presence of the parent in the imaging room may have elicited an emotional response. It is also not clear how naturalistic paradigms may affect intrinsic functional connectivity compared to rest, especially with children showing rapid developmental changes in brain functional connections (Emerson et al., 2015). However, the latest results have shown that data collected during naturalistic viewing improves functional connectivity-based behavior prediction compared to data collected at rest (Finn and Bandettini, 2021).

In conclusion, we found that the quality of mother–infant interaction, more specifically maternal sensitivity, during infancy is associated with local functional connectivity of the mPFC in 5-year-olds. We did not find support for similar associations in the patterns of mPFC connectivity to the rest of the brain, nor did we find significant associations between maternal sensitivity in toddlerhood and functional connectivity. These results imply that variation in maternal sensitivity in infancy may influence child brain functional connectivity in regions related to self-regulation.

## Data availability statement

The datasets presented in this article are not readily available because the ethics committee decision and local legislation do not allow the open sharing of neuroimaging data. Requests to access the datasets should be directed to the corresponding author AC, anmaco@utu.fi.

## Ethics statement

The studies involving human participants were reviewed and approved by the Joint Ethics Committee of the University of Turku and the Hospital District of Southwest Finland. Written informed consent to participate in this study was provided by the participants’ legal guardian/next of kin.

## Author contributions

AC: data acquisition, data analyses, and writing—original draft. SN, JT, and RK: funding acquisition, conceptualization, supervision, and writing—review and editing. OR, EP, VK, ESi, ESa, HH, EH, E-LK, SH, RP, and TL: writing—review and editing. LK and HK: resources, funding acquisition, conceptualization, and writing—review and editing. All authors contributed to the writing of the article and approved the final version.

## Footnotes

1
http://www.nitrc.org/projects/artifact_detect

2
http://rfmri.org/DPABI

3
https://www.fil.ion.ucl.ac.uk/spm/software/spm12
